# Renal Hemodynamic and Functional Changes in Patients with ADPKD

**DOI:** 10.34067/KID.0000000000000412

**Published:** 2024-03-21

**Authors:** Ryota Ishii, Hirayasu Kai, Kentaro Nakajima, Takuya Harada, Tomoki Akiyama, Eri Okada, Ryoya Tsunoda, Toshiaki Usui, Kaori Mase, Naoki Morito, Chie Saito, Joichi Usui, Kunihiro Yamagata

**Affiliations:** 1 Department of Nephrology, Institute of Medicine, University of Tsukuba, Tsukuba, Japan; 2 Department of Nephrology, Ibaraki Clinical Education and Training Center, University of Tsukuba Hospital, Kasama, Japan; 3 Department of Nephrology, Ibaraki Prefectural Central Hospital, Kasama, Japan

**Keywords:** GFR, polycystic kidney disease, renal hemodynamics, renal progression

## Abstract

**Key Points:**

- The mechanism of decreased renal function in autosomal dominant polycystic kidney disease has not been elucidated yet.
- The presented data highlight specific renal hemodynamic changes that occur in patients with autosomal dominant polycystic kidney disease.

**Background:**

Although the mechanisms underlying cyst enlargement in autosomal dominant polycystic kidney disease (ADPKD) are becoming clearer, those of renal dysfunction are not fully understood. In particular, total kidney volume and renal function do not always correspond. To elucidate this discrepancy, we studied in detail glomerular hemodynamic changes during ADPKD progression.

**Methods:**

Sixty-one patients with ADPKD with baseline height-adjusted total kidney volume (Ht-TKV) of 933±537 ml/m and serum creatinine of 1.16±0.62 mg/dl were followed for 2 years. GFR and renal plasma flow (RPF) slopes were calculated from inulin clearance (C_in_) and para-aminohippuric acid clearance (C_PAH_), respectively, while glomerular hydrostatic pressure (P_glo_), afferent resistance (R_A_), and efferent resistance (R_E_) were estimated using the Gomez formulas. Each parameter was compared with baseline Ht-TKV. Patients were also subclassified into 1A–1B and 1C–1E groups according to the baseline Mayo imaging classification and then compared with respect to GFR, RPF, filtration fraction, and glomerular hemodynamics.

**Results:**

After 2 years, Ht-TKV increased (933±537 to 1000±648 ml/m, *P* < 0.01), GFR decreased (66.7±30 to 57.3±30.1 ml/min per 1.73 m^2^, *P* < 0.001), and RPF decreased (390±215 to 339±190 ml/min per 1.73 m^2^, *P* < 0.05). Furthermore, P_glo_ was decreased and R_A_ was increased. Baseline Ht-TKV was inversely correlated with GFR (*r*=−0.29, *P* < 0.05), but there was no association between baseline Ht-TKV and RPF, P_glo_, R_A_, or R_E_ annual changes. However, despite an increase in R_E_ in the 1A–1B group, R_E_ was decreased in the 1C–1E group. As a result, R_E_ slope was significantly lower in the 1C–1E group than the 1A–1B group over time (−83 [−309 to 102] to 164 [−34 to 343] dyne·s·cm^−5^, *P* < 0.01).

**Conclusions:**

This is the first report examining yearly changes of GFR (inulin), RPF (para-aminohippuric), and renal microcirculation parameters in patients with ADPKD. Our results demonstrate that GFR reduction was caused by R_A_ increase, which was faster because of R_E_ decrease in patients with faster Ht-TKV increase.

## Visual Abstract

**Figure:**
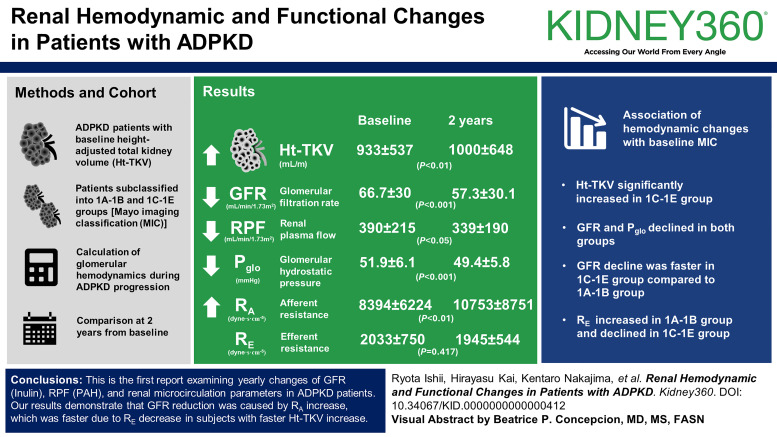

## Introduction

Autosomal dominant polycystic kidney disease (ADPKD) is known as a frequently inherited renal disease in which renal function declines as renal cysts enlarge. In the very early stages of ADPKD, renal hemodynamic changes in the nephron, such as increased filtration fraction (FF) due to hyperfiltration and activation of the renin-angiotensin system, have been suggested.^1,2^ Ischemia of the kidney associated with the enlargement of renal cysts and inflammation of the interstitium have also been implicated in GFR reduction.^3,4^

Although some reports have assessed GFR and renal plasma flow (RPF) using eGFR and radioisotopes in patients with ADPKD, there have been no reports on the changes in GFR and RPF as shown by inulin clearance (C_in_) and para-aminohippuric acid (PAH) clearance (C_PAH_), respectively, over time with enlargement of renal cysts. One suggested mechanism for GFR reduction is renal venous hypertension, which leads to renal congestion.^5^ However, there are insufficient reports on the hemodynamic changes caused by renal cyst enlargement, including glomerular hemodynamic parameters such as glomerular hydrostatic pressure (P_glo_), afferent resistance (R_A_), and efferent resistance (R_E_).

We investigated changes in renal hemodynamics over time by measuring C_in_ and C_PAH_ to determine GFR and RPF, respectively, and simultaneously estimating P_glo_, R_A_, and R_E_ by applying the Gomez formulas. The purpose of this study was to determine the mechanism of GFR reduction associated with cystic enlargement by examining specific renal hemodynamic changes that occur in patients with increased kidney volume in ADPKD.

## Methods

### Patients

Sixty-one patients were entered in this study performed at the University of Tsukuba Hospital from October 2014 to November 2020 and underwent simultaneous measurements for C_in_ and C_PAH_ at approximately 2-year interval. ADPKD was diagnosed based on the Pei classification and the Japanese Ministry of Health, Labor, and Welfare's Guidelines for the Diagnosis and Treatment of ADPKD (second Edition).^6^

### Measurements and Definitions

Total kidney volume (TKV) was measured from abdominal CT using the high-speed 3D image analysis system, SYNAPSE VINCENT (Fuji Medical Systems, Tokyo, Japan). Height-adjusted TKV (Ht-TKV) was calculated by dividing TKV by height (m). GFR and RPF were obtained by measuring C_in_ and C_PAH_ as follows. First, inulin (INULEAD Injection, Fuji Yakuhin, Saitama, Japan) and PAH (SODIUM PARA-AMINOHIPPURATE Injection 10%, Alfresa Pharma, Osaka, Japan) were adjusted to 1% and 0.3%, respectively, with saline. Then, each patient drank 500 ml of water before starting the test, after which blood was drawn, and a continuous infusion of both inulin and PAH at 300 ml/h was administered. Precisely 30 minutes later, the infusion rate was reduced to 100 ml/h, and the patient was encouraged to urinate and drink water as needed. Serum and urinary inulin concentrations were measured by an enzymatic method (Daiyakara Inulin, TOYOBO, Tokyo, Japan), and PAH concentration was measured by a photometric method (PAH Assay Kit, Sigma-Aldrich, Darmstadt, Germany). The mean of three clearances was taken. Body surface area was calculated using the Du Bois formula (weight [kg]^0.425^×height [cm]^0.725^× 0.007184), and GFR and RPF were expressed as per 1.73 m^2^. FF was calculated as GFR/RPF. Body mass index was calculated as weight (kg)/height^2^ (m^2^). Patients were considered treated for hypertension if they were already taking regular antihypertensive medication at the time of the first GFR and RPF measurements. Urinary protein was defined as urinary protein concentration in urine at any time (mg/dl)/urinary creatinine (Cr) concentration (mg/dl).

The Gomez formulas were devised in 1951 by Domingo M. Gomez, a physicist and mathematician, and are used to indirectly evaluate glomerular hemodynamics in humans. The filtration pressure across the glomerular capillaries (ΔP_F_), P_glo_, R_A_, and R_E_ can be calculated by using mean BP (MBP), GFR, RPF, hematocrit (Hct), and total protein concentration (TP). Briefly, the Gomez formulas are shown as follows ^7–9^:

$$\Delta P_{F}=\text{GFR}/K_{FG}$$

$$P_{\text{glo}}=\Delta P_{F}+P_{\text{Bow}}+\pi G$$

$$\pi G=5\times \left(C_{M}‐2\right)$$

$$C_{M}=\text{TP}/\text{FF}\times \ln\;\left(1/1-\text{FF}\right)$$

Application of the above formulas shows that K_FG_ (the gross filtration coefficient) is estimated as 0.0812 ml/s·mm Hg for two kidneys, P_Bow_ (the hydrostatic pressure in Bowman's space) is estimated as 10 mm Hg, and πG (the oncotic pressure within the glomerular capillaries) can be obtained from C_M_ (plasma protein concentration within the glomerular capillaries) and calculated from TP and FF.

Furthermore, Ohm's law states that

$$R_{A}=\left(\left(\text{MBP}-P_{\text{glo}}\right)/\text{RBF}\right)\times 1328$$

$$R_{E}=(\text{GFR}/\left(K_{FG}\times \left(\text{RBF}-\text{GFR}\right)\right)\times 1328$$

Application of Ohm's law demonstrates that 1328 is the conversion factor to dyne·s·cm^−5^, while GFR, RPF, and RBF are expressed as ml/s, and MBP is calculated as (2×diastolic BP+systolic BP)/3.

RBF can be calculated from RPF and Hct using the standard formula:

RBF=RPF/(1−Hct)

For changes over time, the following formula was applied:

%Ht−TKV slope (%/year)=(2nd measured Ht−TKV−baseline Ht−TKV)/baseline Ht−TKV×100/measurement period

The slopes of GFR, RPF, P_glo_, R_A_, and R_E_ were calculated using the following:

(2nd measured value−baseline value)/measurement period

### Genomic Analysis

Patients' genomic DNA was extracted from peripheral blood using the QIAamp DNA blood maxi kit (Qiagen Inc., Hilden, Germany). Mutational analyses of *PKD1* and *PKD2* were performed using the next-generation sequence method for whole-genome sequence analysis. Sequence data were analyzed on the basis of the germline short variant discovery (single nucleotide polymorphisms+indels) of the Genome Analysis Toolkit (GATK) Best Practice. That is, the sequence reads were mapped to a human reference genome (hg38) using the Burrows-Wheeler alignment-maximal exact match algorithm.^10^ By using GATK Tools,^11,12^ duplications of reads were removed, and base quality scores were recalibrated. Single nucleotide polymorphisms and short indels of each sample were called, and mutations of all samples were gathered into a vcf file by joint-call. Mutations were filtrated by the GATK's standard filter and the variant quality score recalibration. Mutations were annotated using snpEff,^13^ and mutations in *PKD1* and *PKD2* genes were collected and evaluated.

### Mayo Imaging Classification

The Mayo imaging classification (MIC) divides typical ADPKD into five groups (Mayo image classes 1A–1E) according to age and Ht-TKV to predict renal outcome. ^14^ We further divided the cohort into two groups, MIC 1A–1B and 1C–1E, because of the limited number of cases in groups 1A and 1E (two patients each). Then, these two MIC groups were compared with respect to GFR, RPF, FF, and glomerular hemodynamics.

### Ethics

The study was conducted in compliance with the Declaration of Helsinki and was approved by the Ethics Committee of our hospital (H26-059). Written informed consent was provided by all participants. The study was also published by a clinical trials registry system recognized by the International Committee of Medical Journal Editors (UMIN000014674).

### Statistical Methods

Statistical analyses were performed with Easy R (Saitama Medical Center, Jichi Medical University, Saitama, Japan), which is a graphical user interface for R (The R Foundation for Statistical Computing, Vienna, Austria). More precisely, it is a modified version of R commander designed to add statistical functions. In all analyses, *P* < 0.05 was considered to indicate statistical significance. Parametric variables are presented as mean±SD, and nonparametric variables are presented as median (interquartile range). Comparison of two categorical variables was performed with Fisher's exact test. Correlation coefficients (*r*) were determined by Pearson's correlation coefficient for parametric variables and by Spearman rank correlation coefficient for nonparametric variables. Comparisons of differences were evaluated by Student's *t* test, Mann–Whitney *U* test, paired *t* test, and Wilcoxon signed rank sum test, as appropriate. All analyses were performed as intention to treat.

## Results

### Characteristics of the Patients

Baseline and subsequent data for the 61 patients who underwent two GFR and RPF measurements over time are shown in Table 1. The median time between the two measurements was 2.07 years, 26 (42.6%) were male and 35 (57.4%) were female, and the mean baseline age was 48.4±11.4 years. Genetic mutations were detected in *PKD1* in 45 (73.8%) cases and *PKD2* in 11 (18%) cases and unidentified or not tested in 5 (8.2%) patients. There were 22 patients who started tolvaptan treatment during the observation period. At the start of the study, 42 patients (68.9%) were taking antihypertensive agents, and at the end of the study, 47 patients (77%) were taking them. At baseline, 18 patients (29.5%) were taking a single antihypertensive medication and 24 (39.3%) were taking multiple antihypertensive medications. About 2 years later, 16 (26.2%) and 31 (50.8%) patients were taking single and multiple antihypertensive medications, respectively. Renin-angiotensin-aldosterone system inhibitors (RAASIs) were taken by 39 patients (63.9%) at the start of the study and 46 patients (75.4%) at the end of the study. Seven patients received an increased dose of RAASIs. Three patients had their RAASIs dose reduced or changed to calcium channel blockers (CCBs) because of hyperkalemia. Twenty-one patients (34.4%) were taking CCBs at the start and 30 (49.2%) at the end of the study. Eleven patients had newly prescribed or increased dose of CCBs.

**Table 1:** Baseline and second total kidney volume, GFR, and renal plasma flow measurements

Variables	Entire Cohort *N*=61		
	Measurement Period, yr	2.07 (1.96–2.19)	
	Sex (*No.* Male/Female)	26 (42.6%)/35 (57.4%)	
	Baseline Age, yr	48.4±11.4	
	Genotype, *No.* (%)	*PKD1*; 45 (73.8), *PKD2*; 11 (18), Mutation Unidentified or Not Tested; 5 (8.2)	
	Baseline Data	Second Data	*P* Value
TKV, ml	1556±966	1669±1174	<0.01
Ht-TKV, ml/m	933±537	1000±648	<0.01
GFR, ml/min per 1.73 m^2^	66.7±30.0	57.3±30.1	<0.001
RPF, ml/min per 1.73 m^2^	390±215	339±190	<0.05
FF, %	18.7±5.9	18.0±4.1	0.389
Cr, mg/dl	1.16±0.62	1.44±0.89	<0.001
Urine protein, g/gCre	0.16 (0.09–0.21)	0.14 (0.09–0.26)	0.139
BMI, kg/m^2^	23.6±3.8	23.6±4.1	0.97
TP, g/dl	7.11±0.44	7.03±0.40	0.141
MBP, mm Hg	96±10	96±11	0.846
Hct, %	38.1±4.3	37.7±4.4	0.259
P_glo_, mm Hg	51.9±6.1	49.4±5.8	<0.001
R_A_, dyne·s·cm^−5^	8394±6224	10,753±8751	<0.01
R_E_, dyne·s·cm^−5^	2033±750	1945±544	0.417
Antihypertensive agents, No. (%)	42 (68.9)	47 (77)	0.415
Single medication, No. (%)	18 (29.5)	16 (26.2)	0.84
Multiple medications, No. (%)	24 (39.3)	31 (50.8)	0.275
RAASIs	39 (63.9)	46 (75.4)	0.237
CCBs	21 (34.4)	30 (49.2)	0.142
Diuretics	6 (9.8)	6 (9.8)	1
Others	4 (6.6)	7 (11.5)	0.529
Tolvaptan oral administration	0	22	<0.001

Parametric variables are expressed as mean±SD, whereas nonparametric variables are given as median (25th–75th percentile). Two categorical variables were compared using Fisher's exact test. *P* values were evaluated by paired *t* test or Wilcoxon signed rank sum test, as appropriate. BMI, body mass index; CCBs, calcium channel blockers; Cr, creatinine; FF, filtration fraction; GFR, GFR from inulin clearance; Hct, hematocrit; Ht-TKV, height-adjusted total kidney volume; MBP, mean BP; P_glo,_ glomerular hydrostatic pressure; RAASIs, renin-angiotensin-aldosterone system inhibitors; R_A_, afferent resistance; R_E_, efferent resistance; RPF, renal plasma flow from para-hippuric acid clearance; TKV, total kidney volume; TP, total protein concentration.

### Annual Change in Each Parameter Over 2 Years

TKV (1556±966 to 1669±1174 ml, *P* < 0.01) and Ht-TKV (933±537 to 1000±648 ml/m, *P* < 0.01) increased significantly during the study period of approximately 2 years while GFR (66.7±30 to 57.3±30.1 ml/min per 1.73 m^2^, *P* < 0.001) and RPF (390±215 to 339±190 ml/min per 1.73 m^2^, *P* < 0.05) were significantly decreased (Table 1). With regard to the glomerular hemodynamic parameters, R_A_ was significantly increased (8394±6224 to 10,753±8751 dyne·s·cm^−5^, *P* < 0.01) and P_glo_ was significantly decreased (51.9±6.1 to 49.4±5.8 mm Hg, *P* < 0.001) (Table 1).

### The Relation between the Annual Changes of Each Parameter and Baseline Ht-TKV

**Figure 1:**
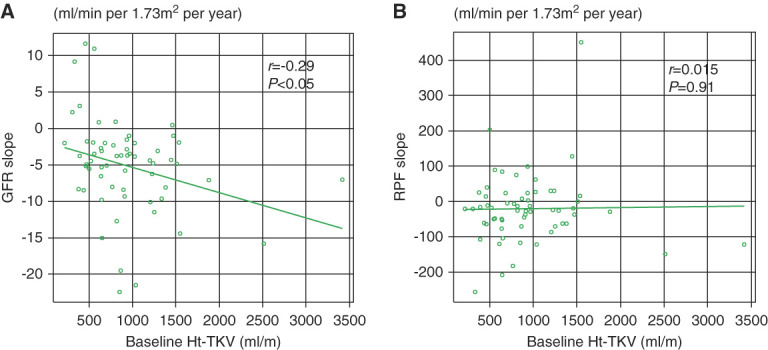
**Relationship between annual change in GFR and RPF and baseline Ht-TKV.** Correlation between (A) baseline Ht-TKV and GFR slope and (B) baseline Ht-TKV and RPF slope. The slope of the reduction in GFR correlated with Ht-TKV, whereas the slope of the reduction in RPF was not correlated with Ht-TKV. GFR, GFR from inulin clearance; Ht-TKV, height-adjusted total kidney volume; RPF, renal plasma flow from para-hippuric acid clearance.

The annual changes of each parameter are shown in Table 2 (left). The mean %Ht-TKV increment speed was 4.08%±8.52%, and the mean GFR and RPF annual changes were −5.08±6.37 and −20.6±98.5 ml/min per 1.73 m^2^, respectively. Furthermore, the mean P_glo_ slope was −1.50±2.68 mm Hg/yr, the median R_A_ annual change was 648 (−219 to 1820) dyne·s·cm^−5^/yr, and the R_E_ annual change was −49 (−269 to 175) dyne·s·cm^−5^/yr. The relations between baseline Ht-TKV and the annual changes in GFR, RPF, and each glomerular hemodynamic parameter are shown in Table 2 (right). The results revealed that baseline Ht-TKV was inversely correlated with the rate of GFR speed (*r*=−0.29, *P* < 0.05) (Figure 1A and Table 2, right) while there was positive, but not significant, relationship between baseline Ht-TKV and Ht-TKV expansion speed (*r*=0.248, *P* = 0.054). Furthermore, there was a negative, but not significant, relationship between baseline TKV and R_E_ (*r*=−0.25, *P* = 0.056). On the other hand, baseline Ht-TKV did not affect the rate of RPF decrease (Figure 1B and Table 2, right). No association was found between baseline Ht-TKV and the slopes of the glomerular hemodynamic parameters, P_glo_, and R_A_.

**Table 2:** Correlation coefficient (*r*) between baseline height-adjusted total kidney volume and slope of each parameter

Slope (Annual Change Rate) Data of Each Parameter		Correlation Coefficient (*r*) Between Baseline Ht-TKV and Each Parameter Slope		
			*r*	*P* Value
%Ht-TKV slope, %/yr	4.08±8.52	%Ht-TKV slope, %/yr	0.248	0.054
GFR slope, ml/min per 1.73 m^2^ per year	−5.08±6.37	GFR slope, ml/min per 1.73 m^2^ per year	−0.292	<0.05
RPF slope, ml/min per 1.73 m^2^ per year	−20.6±98.5	RPF slope, ml/min per 1.73 m^2^ per year	0.015	0.908
P_glo_ slope, mm Hg/yr	−1.50±2.68	P_glo_ slope, mm Hg/yr	−0.151	0.245
R_A_ slope, dyne·s·cm^−5^/yr	648 (−219 to 1820)	R_A_ slope, dyne·s·cm^−5^/yr	−0.016^a^	0.903
R_E_ slope, dyne·s·cm^−5^/yr	−49 (−269 to 175)	R_E_ slope, dyne·s·cm^−5^/yr	−0.25^a^	0.056

*r* is Pearson's correlation coefficient. GFR, GFR from inulin clearance; Ht-TKV, height-adjusted total kidney volume; RPF, renal plasma flow from para-hippuric acid clearance; P_glo_, glomerular hydrostatic pressure; R_A_, afferent resistance; R_E_, efferent resistance.

a Spearman rank correlation coefficient.

### Renal Hemodynamic and Functional Changes and Their Association with Baseline MIC

The number of baseline MIC 1A–1E patients in this study was 2, 18, 22, 17, and 2, respectively. Patients were younger and had a higher rate of *PKD1* mutations in the MIC 1C–1E group compared with the 1A–1B group (Table 3). Baseline parameters including GFR, RPF, FF, and glomerular hemodynamics were not significantly different between 1A and 1B and 1C–1E groups. In the baseline and second comparison, Ht-TKV significantly increased only in 1C–1E group while GFR and P_glo_ declined in both groups. In comparison of changes over time, GFR decline was significantly faster in the 1C–1E group than the 1A–1B group (−6.31±6.85 to −2.57±4.44 ml/min per 1.73 m^2^, *P* < 0.05). On the other hand, R_E_ increased over time in the 1A–1B group (1893±451 to 2141±494 dyne·s·cm^−5^, *P* = 0.089) and decreased over time in the 1C–1E group (2101±855 to 1849±548 dyne·s·cm^−5^, *P* = 0.078). As a result, R_E_ slope was significantly lower in the 1C–1E group than in the 1A–1B group (−83 [−309 to 102] to 164 [−34 to 343] dyne·s·cm^−5^, *P* < 0.01).

**Table 3:** Comparison of each parameter divided into two groups, Group 1A, 1B and Group 1C, 1D, 1E, based on baseline Mayo imaging classification

Variables	Mayo Classification (Entire Cohort *N*=61)		
	1A, 1B (*n*=20) (1A: *n*=2, 1B: *n*=18)	1C, 1D, 1E (*n*=41) (1C: *n*=22, 1D: *n*=17, 1E: *n*=2)	*P* Value
Sex (male/female)	(5/15)	(21/20)	0.06
Baseline age, yr	56.9±11.8	44.3±8.7	<0.001
Genotype (*PKD1*/*PKD2*)	(12/7)	(33/4)	<0.05
Tolvaptan oral administration, No. (%)	4 (20)	18 (43.9)	0.091
RAASIs, No. (%)	15 (75)	31 (75.6)	1
Baseline Ht-TKV, ml/m	573±202	1109±563	<0.001
Baseline GFR, ml/min per 1.73 m^2^	62.8±32.2	68.6±29.1	0.489
Baseline RPF, ml/min per 1.73 m^2^	377±225	396±213	0.742
Baseline FF, %	17.5±3.8	19.3±6.6	0.26
Baseline TP, g/dl	7.15±0.48	7.09±0.43	0.596
Baseline MBP, mm Hg	95±9	97±11	0.63
Baseline Hct, %	37.4±4.3	38.4±4.1	0.399
Baseline P_glo_, mm Hg	50.3±5.1	52.7±6.5	0.154
Baseline R_A_, dyne·s·cm^−5^	9413±6448	7897±6131	0.376
Baseline R_E_, dyne·s·cm^−5^	1893±451	2101±855	0.314
%Ht-TKV slope, %/yr	2.16±8.08	5.01±8.67	0.22
GFR slope, ml/min per 1.73 m^2^ per year	−2.57±4.44	−6.31±6.85	<0.05
RPF slope, ml/min per 1.73m^2^ per year	−30.9±82.8	−15.6±106.0	0.573
P_glo_ slope, mm Hg/yr	−0.78±1.55	−1.85±3.04	0.144
R_A_ slope, dyne·s·cm^−5^/yr	678 (−29 to 3589)	648 (−418 to 1675)	0.332
R_E_ slope, dyne·s·cm^−5^/yr	164 (−34 to 343)	−83 (−309 to 102)	<0.01

Comparison of Baseline and Second Measurement	Baseline	Second	*P* Value	Baseline	Second	*P* Value
Ht-TKV, ml/m	573±202	595±242	0.201	1109±563	1197±693	<0.01
GFR, ml/min per 1.73 m^2^	62.8±32.2	58.2±34.7	<0.05	68.6±29.1	56.9±28.1	<0.001
RPF, ml/min per 1.73 m^2^	377±225	321±218	0.113	396±213	348±177	0.072
FF, %	17.5±3.8	19.3±3.5	0.11	19.3±6.6	17.4±4.3	0.089
TP, g/dl	7.15±0.48	6.95±0.49	<0.05	7.09±0.43	7.07±0.35	0.77
MBP, mm Hg	95±9	95±11	0.787	97±11	97±11	0.67
Hct, %	37.4±4.4	36.6±4.6	0.09	38.4±4.1	38.2±4.2	0.74
P_glo_, mm Hg	50.3±5.1	48.7±5.9	<0.05	52.7±6.5	49.8±5.7	<0.001
R_A_, dyne·s·cm^−5^	9413±6448	12,854±9782	<0.01	7897±6131	9729±8133	0.117
R_E_, dyne·s·cm^−5^	1893±451	2141±494	0.089	2101±855	1849±548	0.078

Parametric variables are expressed as mean±SD, whereas nonparametric variables are given as median (25th–75th percentile). Categorical characteristics comparison was performed with Fisher's exact test. *P* values were evaluated by Student's *t* test, Mann–Whitney *U* test or paired *t* test, as appropriate. FF, filtration fraction; GFR, GFR from inulin clearance; Hct, hematocrit; Ht-TKV, height-adjusted total kidney volume; MBP, mean BP; P_glo_, glomerular hydrostatic pressure; RAASIs, renin-angiotensin-aldosterone system inhibitors; R_A_, afferent resistance; R_E_, efferent resistance; RPF, renal plasma flow from para-hippuric acid clearance; TP, total protein concentration.

### Pretreatment and Posttreatment Changes in Tolvaptan-Treated Patients

For the 22 patients who were administered tolvaptan during the baseline and subsequent measurements, the changes in each parameter are analyzed. TKV and Ht-TKV remained unchanged (1719±776 to 1784±906 ml, *P* = 0.169 and 1032±450 to 1072±530 ml/m, *P* = 0.163, respectively) because of the inhibitory effect of tolvaptan on renal cyst growth. On the other hand, GFR and P_glo_ decreased significantly (53.4±22.6 to 43.6±23.6 ml/min per 1.73 m^2^, *P* < 0.01, and 51.1±5.8 to 47.5±5.3 mm Hg, *P* < 0.001, respectively), although RPF did not change (288±151 to 251±133 ml/min per 1.73 m^2^, *P* = 0.178).

## Discussion

There are various methods for measuring renal function; in addition to eGFR, measured Cr clearance (CCr) and estimated CCr using the Cockcroft-Gault equation can also be used. eGFR may be overestimated in thin elderly people and those with less muscle mass or underestimated when applied to healthy individuals.^15^ Cr is secreted from the tubules in addition to glomerular filtration.^16,17^ Thus, glomerular filtration impairment is positively associated with the contribution of Cr to tubular secretion, and measured CCr is more excessive as compared with GFR as renal function declines.^18^ On the other hand, GFR is considered the gold standard and most accurate measurement of renal function, although the measurement technique is complicated.

There have been various reports on the rate of decline in renal function in patients with ADPKD.^19–21^ The TEMPO 3:4 trial was an interventional trial in patients with an eGFR of 60 ml/min or more.^19^ The annual eGFR worsened by −3.70 and −2.72 ml/min per 1.73 m^2^ in the placebo and tolvaptan intervention groups, respectively. The REPRISE trial is an interventional study in which patients with ADPKD aged 18–55 years with an eGFR of 25–65 ml/min per 1.73 m^2^ or 56–65 years with an eGFR of 25–44 ml/min per 1.73 m^2^ were randomized to tolvaptan or placebo group.^20^ In the REPRISE trial, the rate of eGFR deterioration in the placebo and tolvaptan intervention groups was −3.61 and −2.34 ml/min per 1.73 m^2^, respectively. In Japan, Hoshino *et al.* retrospectively analyzed the annual rate of renal function deterioration in patients with advanced CKD and found that annual eGFR declines in patients with PKD with CKD stages G3b, G4, and G5 were −2.04±0.31, −3.17±0.23, and −3.35±0.29 ml/min per 1.73 m^2^, respectively.^21^ The total annual GFR deterioration rate in this study was −4.54 ml/min per 1.73 m^2^ and −4.83 ml/min per 1.73 m^2^ in tolvaptan group, suggesting that more patients with advanced ADPKD undergoing faster renal function deterioration were included in this study than in previous reports. In addition, because previous studies were based on GFR estimated from serum Cr, it cannot be ruled out that the results may have been influenced by changes in serum Cr levels other than renal function, such as loss of muscle mass over time. This study directly measured accurate GFR in patients with ADPKD over time. Therefore, it may indicate a true decline in GFR.

Comparison of baseline Ht-TKV and %Ht-TKV annual changes showed a trend toward faster increase with larger baseline kidney volume (Table 2, right). The Consortium of Radiologic Imaging Study of PKD study and the Mayo classification suggest an exponential increase in kidney volume in ADPKD.^14,22^ Therefore, a positive correlation was expected between change in TKV and baseline TKV, but the inclusion of tolvaptan-treated patients may have obscured the results. Furthermore, although there was no statistically significant difference in the association between Ht-TKV and %Ht-TKV annual changes in this study, the *P* value was 0.054, which is close to significance considering the rather small sample size. Several previous reports have shown that a larger TKV results in a faster rate of eGFR decline.^22,23^ Similar results were also shown for GFR in this study.

By contrast, no association was found between baseline Ht-TKV and RPF annual change (*r*=−0.015, *P* = 0.908) (Figure 1B). It is known that most PAHs are excreted in the urine by glomerular filtration and secretion into the tubules, thus, RPF reflects the blood flow to the nephrons. Furthermore, in normal kidneys, approximately 10% of the total RPF is through sites not involved in glomerular filtration or tubular secretion. Therefore, the total RPF is calculated as RPF (PAH)/0.9. In ADPKD, as cysts enlarge, electron micrographs show capillary neovascularization and angiogenesis.^3^ Therefore, renal blood flow, which is not assessed by PAH clearance, flows into the paracystic tissue as the cyst expansions. Since the decrease in effective RPF with renal dysfunction in ADPKD is not associated with renal cyst enlargement, this decrease in RPF over time is assumed to be due to changes in renal microcirculation at the level of the nephrons.

Physiologically, factors that define GFR include renal blood flow (*i.e*., RPF) and parameters related to glomerular hemodynamics such as vascular resistance of afferent and efferent arterioles, plasma colloid osmotic pressure, and filtration coefficient of glomerular capillaries.^24,25^ The data presented provide strong evidence that increased renal afferent resistance contributes to decreased GFR in patients with ADPKD. Although it is difficult to strictly identify the contribution of afferent and efferent to renal dysfunction, the results suggest that efferent resistance is also involved in severe cases. One possible mechanism for this finding is that the rapid progression leads to increased angiogenesis in the tubulointerstitial tissue, which generates novel blood flow pathways, resulting in R_E_ reduction, sustained decrease in P_glo_, and faster decrease in GFR.

In this study, we also compared data before and after the initiation of tolvaptan in 22 patients. Although we observed a decrease in GFR during the current 2-year tolvaptan treatment period, maintenance of RPF may have prevented tubular epithelial ischemia and maintained tubular function, which may reflect tolvaptan's long-term effect in maintaining renal function.

There are several limitations to this study. First, the P_glo_, R_A_, and R_E_ calculated by the Gomez formula were simply estimations. However, direct measurement of these glomerular hemodynamic parameters in patients with ADPKD is invasive and technically impossible. Consequently, these are the only methods to estimate and study glomerular hemodynamic parameters in patients with ADPKD. Second, the selection of tolvaptan treatment was made in a real clinical setting, making detailed comparisons between the tolvaptan-treated and nontreated groups impossible. Finally, we did not find statistically significant differences in the association between differences in *PKD1*/*PKD2* gene variant type and renal glomerular hemodynamics, but this could have been due to a small sample size.

In conclusion, our results demonstrate that glomerular hemodynamic changes are one mechanism of GFR reduction in patients with ADPKD. Furthermore, tolvaptan suppresses renal cyst expansion and maintains postglomerular blood flow, which may provide long-term renoprotection by preventing tubular ischemia.

## Data Availability

All data are included in the manuscript and/or supporting information.
